# Estimates of live-tree carbon stores in the Pacific Northwest are sensitive to model selection

**DOI:** 10.1186/1750-0680-6-2

**Published:** 2011-04-10

**Authors:** Susanna L Melson, Mark E Harmon, Jeremy S Fried, James B Domingo

**Affiliations:** 1 Forest Science Department, Oregon State University, 321 Richardson Hall, Corvallis OR USA 97331-5752; 2 Portland Forestry Sciences Laboratory, P.O. Box 3890, Portland OR USA 97208-3890; 3 Green Code LLC, P.O. Box 3232, Las Cruces NM USA 88003

## Abstract

**Background:**

Estimates of live-tree carbon stores are influenced by numerous uncertainties. One of them is model-selection uncertainty: one has to choose among multiple empirical equations and conversion factors that can be plausibly justified as locally applicable to calculate the carbon store from inventory measurements such as tree height and diameter at breast height (DBH). Here we quantify the model-selection uncertainty for the five most numerous tree species in six counties of northwest Oregon, USA.

**Results:**

The results of our study demonstrate that model-selection error may introduce 20 to 40% uncertainty into a live-tree carbon estimate, possibly making this form of error the largest source of uncertainty in estimation of live-tree carbon stores. The effect of model selection could be even greater if models are applied beyond the height and DBH ranges for which they were developed.

**Conclusions:**

Model-selection uncertainty is potentially large enough that it could limit the ability to track forest carbon with the precision and accuracy required by carbon accounting protocols. Without local validation based on detailed measurements of usually destructively sampled trees, it is very difficult to choose the best model when there are several available. Our analysis suggests that considering tree form in equation selection may better match trees to existing equations and that substantial gaps exist, in terms of both species and diameter ranges, that are ripe for new model-building effort.

## Background

The rapid increase in atmospheric carbon dioxide (CO_2_) concentration is a major contributor to primarily anthropogenic global warming [1]. International agreements such as the Kyoto Protocol require participating nations to reduce CO_2 _and other greenhouse gas emissions. To implement such commitments, countries must produce nation wide inventories of carbon (C) sources and sinks. Forests can be both C sources and sinks, so there is interest in exploring forest C sequestration to offset anthropogenic CO_2 _emissions [2]. However, before sequestration potential can be assessed, the magnitude of forest C sources and sinks must first be determined.

Live trees are a significant C storage pool in United States of America (US) forests, ranking second behind soil C [3,4]. Live-tree C is often estimated from regression equations that relate biomass (or volume subsequently expressed as biomass using density conversion factors) to some easily measured tree dimension obtained from inventory data, such as DBH (diameter at breast height, usually 1.37 m above ground level) or height. Estimated biomass is then converted to C with a C:biomass ratio (e.g., [3]).

Estimates of C in live trees are influenced by numerous uncertainties: sampling error associated with the inventory (affects precision but not bias as long as the sampling is well designed); measurement uncertainty (can affect both precision and bias); regression uncertainty inherent in any estimated regression relationship (usually affects only precision unless fit is poor) [5]; and model-selection uncertainty (can affect both precision and bias) introduced by having to choose among multiple, potentially equally applicable regression relationships and conversion factors. Each source contributes to uncertainty about the live-tree C estimate (uncertainty in the sense of Harmon et al. [6]). Sampling error and measurement uncertainty are typically studied and addressed by those taking inventories (e.g., [5,7]), regression uncertainty is often assessed by those who publish regression equations (typically R^2 ^and mean standard error are reported, e.g., [8]). Model selection uncertainty is rarely considered, although some authors have noted large differences between prediction equations, e.g., [9-11]. The first three types of uncertainty are routinely assumed to be independent for each sampling unit or individual tree in an inventory, a convention that assists in their estimation and results in minimal aggregate uncertainty when large numbers of trees are inventoried [5]. However, both regression and measurement uncertainty can have a substantial bias component. Model-selection uncertainty, when it occurs, is systematic error, and cannot be modeled independently for individual trees.

To quantify and better understand the uncertainty that selection among models could contribute to regional live-tree C estimates, we conducted a sensitivity analysis on the model-selection component involved in estimating live-tree C for a subset of tree species in northwest Oregon (NWOR), USA. While this is an example from a single region, the findings have relevance to forests globally. For each species we calculated the range of live-tree total C estimates for each size class. These ranges were then applied to inventory data to estimate the range of model-selection uncertainty of live-tree C stores for the study area. Finally, we examined several strategies to reduce this uncertainty in live-tree C estimates.

## Methods Overview

Procedures for this sensitivity analysis were iterative, required a number of assumptions, and as this was a novel approach, necessitated the introduction of nonstandard terms. Distilled to the essence, we: (1) selected the most common tree species in the study area as the population of interest with respect to live-tree carbon estimation; (2) obtained candidate equations, then set and applied criteria to select equations for inclusion; (3) estimated height for each DBH class for each species for DBH-and-height equations; (4) created a calculation "road map" for combining tree parts to generate total tree estimates; (5) computed a range of predictions, which we call prediction envelopes; (6) devised 3 calculation approaches to test sensitivity to alternative assumptions about the acceptability of extrapolating equations beyond the DBH range used in their development (hereafter termed the developmental range); (7) selected a biomass-to-C conversion factor; (8) incorporated alternative assumptions about "correlations" among different tree components; (9) created total live-tree C estimates for each DBH class for each species and applied the resulting ranges to inventory data to produce live-tree C estimates for the study area; and (10) explored alternatives for reducing model selection uncertainty. Detailed methods appear after the Conclusions.

## Results

### Tree-level uncertainty

**Figure 1:**
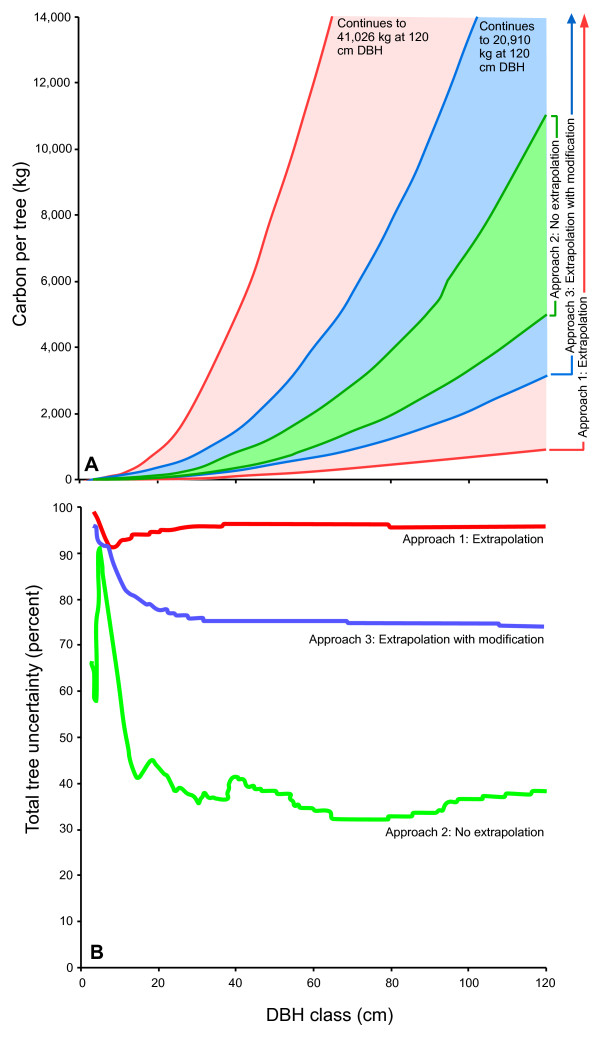
**Percent uncertainty by diameter at breast height (DBH) class and approach**. A. Total live-tree C prediction envelopes by approach for *Pseudotsuga menziesii *(positive correlation). B. The prediction envelopes, displayed as percent uncertainty (half the width of the prediction envelope expressed as a percentage of the midpoint at each DBH class) for total live-tree C, *Pseudotsuga menziesii*, by approach for positive correlation only. Abrupt changes in uncertainty occurred where plots of equation predictions crossed, where an equation began or ended, and occasionally when corrections were applied (in the case of approach 3 only).

Given that there are often multiple regression models for individual tree components, and these components can be combined in multiple ways, we expected, and found, a range of predictions of total live-tree biomass for any species-DBH class. The prediction envelopes we created from the multiple regression equations available for NWOR species indicate that model-selection uncertainty expressed as a percentage of the midpoint varied somewhat across all DBH classes and was considerable for the total tree component, even when predictions were strictly limited to the developmental range of the models (approach 2; Figure 1). This indicates that the wide range of possible biomass values was not solely a function of extrapolation.

**Figure 2:**
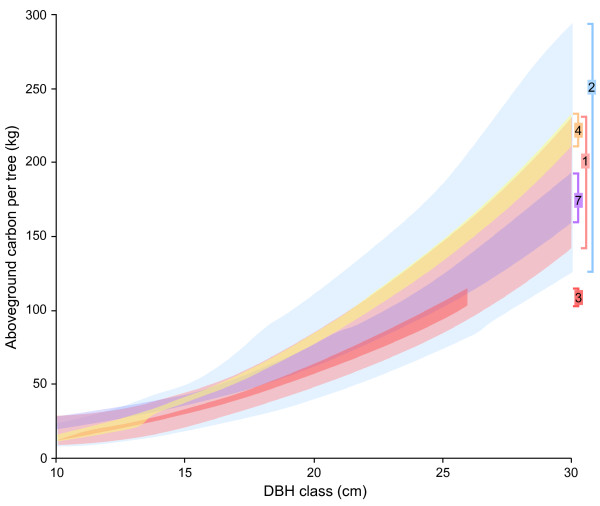
**Comparison of predictions from different calculation pathways**. *Pseudotsuga menziesii *prediction envelopes for a component derived from different sets of equations often overlap considerably. Shown here is the overlap of aboveground carbon between 10- and 30-cm diameter at breast height (DBH) classes under approach 2 with positive correlation. This DBH range allows better visualization of envelope overlap than the full 3-66 cm range used in the analysis. Numbers on the right correspond to the numbered aboveground total prediction envelopes from calculation pathways in Figure 6.

Although substantial uncertainty was indicated for many components by most prediction envelopes, there was a fair degree of consistency between prediction envelopes for the same component derived from different calculation pathways (Figure 2). This indicates that this form of uncertainty is not especially sensitive to how tree components are divided.

**Figure 3:**
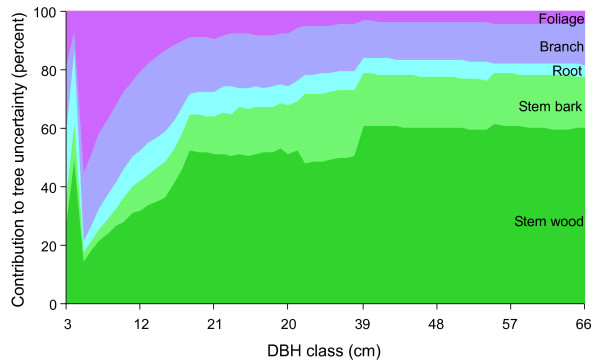
**Contribution of component uncertainty to total tree carbon uncertainty**. Contribution of stem wood, stem bark, coarse roots, total branch, and total foliage uncertainty to total tree uncertainty for *Pseudotsuga menziesii*, approach 2, positive correlation. Stem wood was the greatest contributor to total tree uncertainty at most diameter at breast height (DBH) classes for all species. Note that total tree uncertainty derived in this way was not always equal to the total tree uncertainty calculated using the roadmap (Figure 6) because the input components were derived from only one of the possible calculation pathways.

Stem wood proved to be the most massive component (among stem wood, stem bark, coarse roots, branches, and foliage). It was also the greatest contributor to total tree uncertainty for almost all DBH classes (Figure 3). Coarse roots were often second to stem wood in magnitude, but not in their contribution to uncertainty. This is likely due to a dearth of root equations with large developmental DBH ranges.

As expected, assumption of positive correlation between tree components produced wider total live-tree C prediction envelopes than did negative correlation assumptions. For *Pseudotsuga menziesii*, using approach 2, average percent uncertainty over the 3 to 66 cm DBH range was 38% for positive correlation, but this was reduced to 23% with negative correlation. In general, using negative correlation assumptions halved the average percent uncertainty.

**Figure 4:**
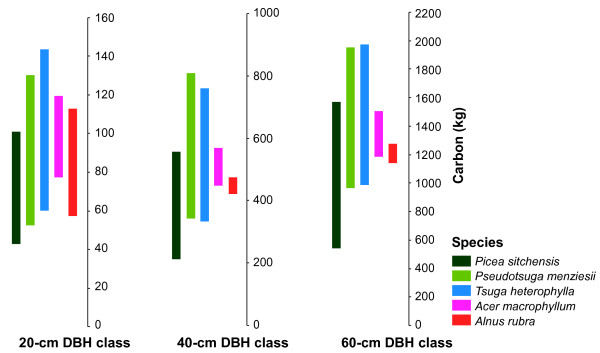
**Total tree C prediction envelope widths for the target species at three diameter at breast height (DBH) classes**. Shown here is output from approach 2 with positive correlation. Narrow ranges do not necessarily indicate better agreement between equations; because approach 2 limited equation use to the developmental DBH ranges, narrow ranges usually indicate a scarcity of equations for a DBH class. Species in the chart for each diameter class appear in the same order as listed in the legend.

Total tree prediction envelopes were remarkably similar among species, indicating that uncertainty related to model selection was a general phenomenon (Figure 4). For approach 2, *Pseudotsuga menziesii *and *Tsuga heterophylla *had similar envelopes and *Acer macrophyllum *and *Alnus rubra *appeared similar as well, and both deciduous tree envelopes were encompassed by the *Pseudotsuga menziesii *and *Tsuga heterophylla *ranges. The *Picea sitchensis *prediction envelope had the lowest lower bound, and its prediction envelope ceased to overlap with other softwood species at about 80 cm DBH, after the endpoint of a set of higher-predicting equations. *Picea sitchensis *envelope width over the 3 to 66 cm DBH range was very similar to that of the other softwoods, just shifted downward, perhaps reflecting a shorter growth habit or narrower upper trunk.

### NWOR live-tree uncertainty

Live-tree carbon stores were highly sensitive to model selection regardless of the component correlation assumption or the manner in which equation extrapolation was handled (i.e., approach1: extrapolation, approach 2: no extrapolation, approach 3: extrapolation with modification). Applying prediction envelopes to the subset of NWOR trees for which we had sufficient equations (target species 3 to 66 cm DBH) indicated estimates of live tree C stores would fall between 26 and 512 Teragrams (Tg; 1 Tg = 1 × 10^12 ^g) C (91% uncertainty relative to the midpoint estimate) for approach 1, between 56 and 119 Tg C (36% uncertainty) for approach 2, and between 36 and 193 Tg C (68% uncertainty) for approach 3 (Table 1) assuming a positive correlation. Approach 1 was expected to inflate uncertainty, yet uncertainty remained high even when either extrapolation was not undertaken (approach 2) or when corrections were applied (approach 3). Negative correlation assumptions reduced uncertainty relative to the midpoint estimate to 66, 20, and 37% for approaches 1, 2, and 3, respectively. Approaches 1 and 3 were sensitive to the inclusion of extreme-predicting equations, e.g., removal of 9 equations developed for seedlings/small saplings that resulted in extreme estimates when extrapolated produced uncertainties of 70% and 54% (approach 1 and approach 3, respectively) for positive correlation and 31% and 26% for negative correlation.

**Table 1:** Live-tree carbon (C) ranges and uncertainty for northwest Oregon (NWOR)

Positive correlation	Trees 3 to 66 cm diameter at breast height (DBH)								
	Approach 1: No corrections			Approach 2: Developmental DBH range			Approach 3: With corrections		
	
Species	Minimum	Maximum	Uncertainty	Minimum	Maximum	Uncertainty	Minimum	Maximum	Uncertainty
	Tg C	Tg C	% of midpoint	Tg C	Tg C	% of midpoint	Tg C	Tg C	% of midpoint
*Picea sitchensis*	1.54	4.75	51	1.53	4.13	46	1.55	4.39	50
*Pseudotsuga menziesii*	9.56	455.32	96	31.26	70.40	38	18.57	135.57	76
*Tsuga heterophylla*	10.54	32.11	51	11.40	26.33	40	10.99	30.57	47
*Acer macrophyllum*	1.48	3.12	36	1.92	2.76	18	1.83	3.13	26
*Alnus rubra*	2.40	16.19	75	10.13	15.59	20	2.98	19.40	73

NWOR total	25.53	512.21	91	56.43	119.19	36	35.92	193.26	68

**Negative correlation**	**Trees 3 to 66 cm diameter at breast height (DBH)**								
	**Approach 1: No corrections**			**Approach 2: Developmental DBH range**			**Approach 3: With corrections**		
	
**Species**	**Minimum**	**Maximum**	**Uncertainty**	**Minimum**	**Maximum**	**Uncertainty**	**Minimum**	**Maximum**	**Uncertainty**

	Tg C	Tg C	% of midpoint	Tg C	Tg C	% of midpoint	Tg C	Tg C	% of midpoint
*Picea sitchensis*	2.26	3.61	23	1.93	3.37	27	2.13	3.57	25
*Pseudotsuga menziesii*	28.30	215.23	77	38.18	60.68	23	38.47	98.05	44
*Tsuga heterophylla*	15.58	23.97	21	14.07	21.08	20	14.93	23.92	23
*Acer macrophyllum*	1.90	2.70	17	2.08	2.63	11	2.16	2.78	13
*Alnus rubra*	5.10	14.40	48	11.85	14.08	9	7.78	14.40	30

NWOR total	53.05	259.91	66	68.11	101.83	20	65.48	142.73	37

Note: Results of applying species tree total prediction envelopes to the Forest Inventory and Analysis database for NWOR. Uncertainty is half of the carbon range, expressed as a percentage of the range midpoint.

Species contribution to NWOR uncertainty was closely correlated with the estimated number of trees of the species (as calculated in the Forest Inventory and Analysis (FIA) Integrated Database [12]), and even more closely to the NWOR live-tree C midpoint of each species. Prediction envelopes were not that different among species, so species contribution to NWOR uncertainty followed species prevalence in the inventory. Under approach 2, *Pseudotsuga menziesii *accounted for 62%, *Tsuga heterophylla *24%, *Alnus rubra *8%, *Picea sitchensis *4%, and *Acer macrophyllum *1% of NWOR live-tree C uncertainty, whereas they accounted for 58, 22, 15, 3, and 3% of the estimated live-tree C (at the midpoint of the range) for the same species (percents may not sum to 100 due to rounding). *Pseudotsuga menziesii*, therefore, contributed slightly more uncertainty to NWOR live-tree C than expected, and *Alnus rubra *contributed less.

### Comparison with other Pacific Northwest (PNW) regional estimates

The first comparison with single-source total live-tree C estimates for NWOR *Pseudotsuga menziesii *26 to 60 cm DBH (see complete Methods for details) demonstrated that these particular single-source estimates clustered around the midrange of our positive correlation approach 2 output (25 to 55 Tg). Equations from Gholz et al. [8] predicted 38, Jenkins et al. [13] 39, Harmon et al. [14] 40, Grier and Logan [15] 41, Shaw [16] 42 Tg C, and the FIA-based estimate predicted 37 to 42 Tg C (positive correlation). The range spanned by single-source estimates covered 15% of our prediction envelope range. However, the more single-source estimates that were included, the wider the range of single source estimates became. Comparison of estimated aboveground total live-tree C (8 single-source predictions) for the same trees produced a range that spanned 43% of our output range, whereas stem wood plus bark live-tree C (11 single-source estimates) yielded a range that covered 53% of ours.

The second comparison using all species produced an FIA-based estimate of 81 to 99 Tg C and a Jenkins et al. [13] estimate of 83 Tg C. Our approach 2 positive correlation assumption generated a range of 56 to 119 Tg C.

### Comparison with other estimates of error

The estimated 95% confidence interval for sampling error from the FIA inventory was roughly +/-6% of the C estimate for NWOR. Measurement error in DBH, treated as a normally-distributed error, introduced 0.03% uncertainty into the FIA stem wood volume estimate. The range created from reported Jenkins et al. [13] 80% of residuals bounds was 63 to 105 Tg C, corresponding to 25% uncertainty.

### Strategies to reduce model-selection uncertainty

#### Comparison of Equation Forms

Our comparison of height-diameter-based equations with diameter-based equations for *Pseudotsuga menziesii *suggested that incorporation of height did not produce greater agreement among predictions. By this test, DBH-height equations appeared no more universally-applicable than DBH-only forms, nor did they appreciably decrease uncertainty related to model selection. This is in agreement with the findings of others [10,11,17].

#### Assigning Equations to Subpopulations

When prediction envelopes were subdivided and trees assigned among them, uncertainty was reduced proportionally to the number of divisions; i.e., dividing the envelope in two halved the uncertainty and dividing the envelope into 10 sections resulted in one-tenth the uncertainty obtained when using the full-width prediction envelope. This suggests that if one could correctly assign biomass equations within species, one could greatly reduce this form of uncertainty.

## Discussion

We examined the sensitivity of live-tree carbon estimates to model selection. Rather than use a single model to estimate total tree carbon, we used multiple total tree models as well as tree component models, the results of which were summed via multiple pathway permutations to estimate total tree biomass and ultimately, carbon. Although some might regard estimating tree level carbon by adding up multiple, modeled components as an unlikely way to attempt total tree C estimation, in practice, analysts such as those at PNW FIA who rely on BIOPAK [18] and other equation compilations frequently do assemble estimates by combining component estimates, sometimes because the developmental DBH range of available total tree equations is more limited than the ranges for component equations or because the sample size for some component equations (e.g. bole volume) is much greater than for other components. Even then, there are unavoidably some trees in the sample that are larger than the developmental ranges of the equations used for a given species and location.

We found that the range of estimates was quite large at the level of tree components, total trees, and NWOR. The uncertainty introduced by selecting different models was high regardless of species or how tree components were combined (either in terms of subcomponents or the type of correlation of tree components).

Given that extrapolation is often required, we considered several approaches, each with advantages and disadvantages. Approach 1 made no assumptions while retaining many predictions for every DBH class; however, this created difficulties by incorporating extreme equation behavior into prediction envelopes. Approach 2 largely removed such problematic equation behavior; however, at small and large DBHs there was often just one applicable equation, which probably resulted in an artificial uncertainty reduction caused by the narrow prediction envelope. Furthermore, component equations were only available to predict tree total C for a small range of NWOR DBHs, and this resulted in our being able to only compare approaches between 3 and 66 cm DBH. Approach 3 generated what appeared to be more realistic prediction envelopes than approach 1, but relied on an extrapolation approach based on modelers' assumptions of acceptable equation behavior.

Comparison of NWOR live-tree C estimates from approaches 1 and 2 (Table 1) reinforces the too-infrequently-heeded warning against equation extrapolation. Uncertainties of 90% for approach 1 over the 3- to 66-cm DBH range, where 81% of the target species trees in NWOR occur, are unacceptable when attempting to balance the global C budget or calculate C credits. Short of conducting studies to create more biomass prediction equations, some extrapolation is inevitable, however. Realistically, it is unlikely that uncertainties of this magnitude exist in current biomass or C estimates because approach 1 included equations so obviously unsuited to estimation at DBHs outside their developmental DBH ranges that they would be discarded by researchers during analysis. Note that although equations which predicted negative values were not excluded from approach 1 unless they were lacking developmental DBH range metadata, very few equations produced negative predictions between 3 to 66 cm.

It seems reasonable to suppose that equations with developmental DBH ranges that lie far from a target DBH class will be worse predictors than those with developmental DBH ranges that span the target DBH class or classes of interest. We explored this by selecting three DBH classes (20, 60, and 100 cm), then finding equations with developmental DBH ranges that (1) spanned the DBH class, (2) ended at half the DBH class, or (3) started at twice the DBH class. We then predicted biomass at the selected DBH class using equations from each available category and determined that equations with developmental DBH ranges distant from the target DBH classes produced wider ranges of estimates, with midpoints shifted from those produced by the equations that spanned the given DBH class. This further illustrates that equation extrapolation generates additional uncertainty.

Although prediction envelopes indicated wide C ranges for large-DBH trees, large-tree percent uncertainty was not necessarily higher; in many cases it was less than for very small trees. Even though it initially appears that creating large tree equations might be the most useful way to reduce uncertainty, that may not be the case. When considering how best to reduce uncertainty from model selection, the underlying NWOR DBH distribution should also be considered. Currently most NWOR C is found in trees between 20 and 70 cm DBH, and large trees are rare. Therefore, a more practical way to reduce uncertainty would be to better identify how to assign these mid-range trees to an appropriate equation. However, for areas where the DBH distribution is shifted toward larger DBHs, extrapolation would introduce more uncertainty in aggregated totals, and investment in determining better-predicting equations would be more worthwhile.

### Comparison with other PNW regional estimates and estimates of error

To evaluate various regional estimates of live C stores, one would ideally compare not only the mean estimate, but also the uncertainty bounds [6]. Unfortunately few studies have produced the latter, and even when this is the case some key components contributing to uncertainty have not been considered. We previously presented two alternative estimates to provide context and points of comparison for our estimates: FIA-predicted biomass [12] and Jenkins et al. [13] general biomass equations.

Both of these estimates were consistent with outputs from this study. Our estimate included only model-selection uncertainty, and the FIA estimate included sampling error that contributed approximately 6% uncertainty (plus limited model-selection uncertainty introduced by our C:biomass conversion factor range used on our foliage, dead branch, and coarse root prediction envelopes). The fact that our midpoint estimates are similar reflects that FIA-selected equations for many species were near the midpoint of target species component prediction envelopes.

Jenkins et al. [13] 80% bands derived from pseudodata residuals predicted a similar range to our approach 2 positive correlation range. This is hardly surprising given that our approach 2 bears great resemblance to their procedure, except they determined a central tendency whereas we retained the bounds. Applying their 80% regression-residual bounds to their estimate is essentially re-building the bounds of the equations they incorporated. The equations in [13] are simple to apply and are national in scope; consequently they may be widely used for estimation. For four of the NWOR target species as well as the NWOR total, these equations produced midpoints and ranges similar to those in our study. Estimates for *Picea sitchensis*, however, were considerably higher in all our approaches. This highlights that care must be taken to determine how well national biomass estimators predict at a regional level.

### Relative uncertainty of error components

When estimating uncertainty in biomass and C estimates, at least four types of errors/uncertainties need to be considered: measurement, sampling, regression, and model selection. Of the four, the first three are best understood. Because they are usually modeled as random errors, region wide estimates of error are very low. Phillips et al. [5] considered sampling, regression, and measurement errors in FIA volume estimates. Given that they considered only one equation for softwoods and another for hardwoods, they did not address what we term model-selection uncertainty. They determined that measurement error was the smallest error component, accounting for only 0.1% of the overall variance (from the three factors). Our quick estimate of measurement error in NWOR volume indicated that it was also quite a small contributor to overall live-tree C uncertainty. Phillips et al. [5] found that sampling error was the largest error component, accounting for 98.7% of overall variance. Sampling error calculated for NWOR was similarly much larger than measurement error. The overall standard error from the five southeastern states they studied only amounted to about 0.6% of the total volume estimate. We made no calculations of regression error, but had we calculated standard error for NWOR in the manner of [5], we believe it would be quite low. This calculation method assumed independence between sampling units and/or trees in all cases. However, if even a small amount of systematic error were present, it could yield a large uncertainty when tree volumes were aggregated [19]. Were the large potential systematic errors arising from model selection choice incorporated, we suspect overall uncertainty would increase by at least an order of magnitude.

In estimating live C stores for the US, Heath and Smith [20,21] subjected the FORCARB model to an uncertainty analysis and concluded that uncertainty for total forest C (i.e., live, dead, soil) in U.S. private forests was +/-9% of their 20 petagram (Pg; 1 Pg = 1 × 10^15 ^g) C estimate for the year 2000. Of nine model parameters examined, the tree volume-to-C conversion factor was second only to soil C in its contribution to the overall uncertainty. Our analysis seems relevant to two of their model parameters: volume and the volume-to-C conversion. FORCARB relies on FIA inventory tree volumes, and Heath and Smith [20] used reported FIA estimates of sampling error to arrive at a +/-5% sampling uncertainty estimate for volume (their uncertainty is expressed as a percentage of the median and represents +/-2 standard errors). This is similar to the +/-6% sampling error for volume estimated from our FIA dataset for NWOR. Their volume-to-C conversion factor was assigned +/-15% uncertainty [20]. This approximated the uncertainty of our two-step volume-to-carbon conversion, which had an estimated range of +/-10% of the midpoint (for stem wood averaged across species). Heath and Smith [20] apparently did not include what we term model-selection uncertainty. Our analysis indicated model-selection uncertainty in NWOR for stem wood volume was 12% (from 22 stem wood volume equations, using approach 2 procedures over DBH classes 10 to 40 cm to allow inclusion of all species). The NWOR model-selection uncertainty for stem wood biomass was 22% (calculated from 44 stem wood biomass equations over the same DBH range). Inclusion of this level of model-selection uncertainty into the FORCARB uncertainty analysis would have likely increased tree C uncertainty and total forest C store uncertainty above the +/-9% they reported for their base model, perhaps to the point where it would exceed soil uncertainty [21].

### Representing model-selection uncertainty

Although sensitivity to model selection has rarely been considered when estimating uncertainty in live-tree volume, biomass, or C stores, our analysis indicated it could be the most significant contributor to uncertainty. In our study, we chose to develop prediction envelopes to represent this facet of uncertainty. The advantage of this approach is that no assumptions about the form or weighting of equations need to be made. Given that the input equations were not part of an overall experimental design and that a variety of equation forms were used, prediction envelopes allow one to use the maximum amount of information. A disadvantage of this approach is that information about central tendencies of the calculation pathways is essentially discarded so the characterization of model-selection sensitivity is greater than it would be otherwise. Such approaches are also not amenable to statistical analysis. Furthermore, there are the issues of nonadditivity and back-transformation of log-log equations. Nonadditivity occurs when predictions from component equations do not sum to the prediction from an aggregated component equation developed from the same trees, and until recently [22] developers of biomass equations did not pay it much heed, although it was remarked on by biometricians for years [23,24]. Using a sample of four sets of equations developed for *Pseudotsuga menziesii *that did not appear to have been constrained to ensure additivity, we found that additivity error for aboveground total biomass (over the developmental DBH range) was nowhere greater than 5%, and averaged -0.04, 1.18, -1.46 and 2.06% overall (equations from [16,25,26], and [27], respectively). Back-transformation of log-log equation predictions is a much debated issue, with some pointing out that without such back-transformation, estimates are biased downward [28]. This was true for a subset of natural-logarithm-transformed volume and biomass equations that we examined, where bias as a percentage of the uncorrected values ranged from 0.7% for stem wood to an astounding 153% for dead branches. Mean biases for stem wood were 3.19% and 8.7% for stem bark (16 equations each). Other researchers contend that back-transformation introduces its own set of biases [13]. Our largely uncorrected (in some cases corrections may have been applied by authors, but it wasn't clear) equations may therefore have introduced bias. The ranges of our prediction envelopes, however, were such that we deemed possible additivity and back-transformations biases unremarkable (assuming possible bias of 153% was quite uncommon) and they are unavoidable anyway by anyone using these sets of equations.

Jenkins et al. [13] pursued an alternative approach to dealing with model-selection sensitivity by developing general equations. They presented a set of national biomass equations, grouped by species similarity, that were based on a library of previously published regional and local equations. Lacking the original tree-level data, they created their new equations from pseudodata generated from the equation library. This approach utilizes the central tendency information inherent in the equation library but essentially discards the outer bounds and introduces various problems related to using pseudodata to generate equations [22]. If the general equations truly represent the central tendency, they should consistently predict total live C stores for geographic areas comparable to those on which the library of equations was based. However, the use of the general equation may increase uncertainty, particularly when analysis is aimed at specific species or subregions dominated by particular species. Case and Hall [29], working with boreal forest data from west-Central Canada, determined that local and generalized regional biomass equations provided acceptable site-level estimates but that generalized national equations [22] produced considerably higher average predication errors at the site level. Mean prediction biases from national equation predictions were also statistically different from local and regional ones for 5 of 10 species. It is currently impossible to determine if equations in [13] produce unbiased estimates at regional/national levels (as we have little truth against which to compare estimates); however, when estimating for some regions, such as Ponderosa pine forests in the interior West, using the all-pine equation [13] that is constructed from equations developed for not only *Pinus ponderosa *(Ponderosa pine) but also for the comparatively faster-growing *Pinus taeda *(loblolly pine) and *Pinus elliottii *(slash pine), bias is likely. The difference in C estimates for *Picea sitchensis *between this study and the Jenkins et al.-based estimate [13] indicates potential bias, possibly arising from their grouping of *Picea sitchensis *with other *Picea *that have shorter growth habits and the relative scarcity of *Picea sitchensis *sources compared with those of other *Picea *species (i.e., 2 for *Picea sitchensis *versus 25 sources for 5 other *Picea *species). Bias would be unlikely if the equations were included in proportion to the abundance of tree species and area represented in the area to be analyzed. Inclusion of too many equations over a part of the DBH range or from a particular type of site or species could weight the overall equation in that direction, even if that type were rare.

Our approach and the Jenkins et al. [13] method both relied on existing biomass equations. However, there are major problems with existing equations [9,13,30]. These include inappropriate or nonrepresentative selection of trees in the development of equations, limited sample sizes (especially when large trees or difficult-to-measure components such as roots are involved), and limited sample DBH ranges. Equations (especially for volume) come in a variety of forms, and there is no consistent partitioning of trees into components. Even for a major component such as stem wood, equations differ in assumptions of stump heights and top diameter, complicating comparisons among models. Crowns are notorious for the variability in the approaches to their division into components, with branches classified or grouped at varying diameter breakpoints, foliage either included with the smallest branch class or not, and branches and foliage split into live and dead classes or not. Furthermore, component equations relying on nonlinear transformations of data are nonadditive. Use of different equation forms for different components (unless special procedures are observed during equation development [22]) also contributes to the nonadditivity of component equations [31]. Statistical information necessary to compare equations is rarely presented, and few publications include the necessary information to create regression prediction intervals, so generation of pseudodata representing the true level of variation in predictions is not possible. Data describing site and sample characteristics lack consistency as well, making comparisons among equations based on these characteristics problematic. Raw data are rarely presented, but as Jenkins et al. [13] observed, this would be helpful to researchers developing new generalized equations. Authors of some recent North American equations have borne this in mind and provide, if not data, then at least more complete regression statistics and component equations that are additive [22].

### Reducing uncertainty due to model selection

The considerable expense of developing new biomass equations and the urgency in getting to a system that can accurately characterize forest C stores and flux in support of C management, argues for utilization to the maximum extent practicable the biomass equations and data that have already been developed. Unfortunately these equations and data were typically developed to represent specific geographical areas, ranges of tree sizes, or tree components, and there is no practical way to objectively assess bias of the existing systems of equations. Although it would be unrealistic to set aside all existing equations and begin anew, an effort to more systematically capture the variability present within and between tree species would contribute to understanding the scope of the potential bias and uncertainty introduced by model selection. Such efforts have been untaken in some regions (e.g., manipulations of the Canada ENFOR data [22]) and are a logical starting point.

There are several ways to reduce uncertainty owing to model selection. Our analysis indicates that subdividing biomass equations would reduce uncertainty, but to succeed, development of a consistent and robust method for choosing the best equation for each tree is needed. To some degree, equations can be selected based on geography (e.g., equations for Douglas-fir in coastal versus interior British Columbia [26]). For example, PNW FIA already applies different equations for a quarter of the conifer species in their database depending on whether the tree is located east or west of the Cascades [12]. However, equations developed from stands growing in proximity and apparently similar physiographic situations can also yield differing predictions, although closely matching the DBH range of the target to the developmental population may help in choosing an equation with a good fit [10]. Understanding the degree to which local-scale factors control tree form, and the possible influence of genetics, would contribute to better model selection.

Another approach would be to use a biomass equation that is truly general. Inclusion of height in biomass equations is sometimes thought to create a more widely applicable equation, but our examination of equation predictions for *Pseudotsuga menziesii*, the most-sampled species in the PNW, indicated that there was no more agreement between height-and-DBH-based equations than among DBH-only ones (use of height-and-DBH-based equations might be preferable in limited circumstances, such as managed stands that have not arrived at crown closure [32]). This is probably due to the fact that tree form varies greatly. In the case of excurrent forms (those with a strong central leader), trees forms can range from paraboloids to cones to neiloids. Although few species span this entire range of forms, such differences in tree taper patterns could lead to differences of approximately 50% in volume and biomass between two trees, even when their DBH and height are identical. To some degree, these differences can be accounted for by knowing the species. Inclusion of a form factor into biomass equations may reduce model-selection uncertainties but create other problems. As with height, it would be difficult to determine the form of each tree; therefore, some prediction of form would be required. Moreover, development of efficient ways to quantify form and taper would also be needed, possibly via subsampling mid-height diameters in stands (for excurrent forms) and height to the first major branch (for deccurrent forms - those with weak central leaders). The degree to which the uncertainty introduced by such prediction offsets that introduced by model selection would require further investigation.

## Conclusions

Sensitivity of NWOR live-tree estimates to model selection was substantial at every level examined and varied with the degree of correlation assumed between tree components. Especially considering the potential for this form of uncertainty to introduce bias, it is likely more important than the combined uncertainty introduced via measurement, sampling, and regression. This facet of uncertainty has not been generally appreciated because the full range of available biomass equations has not been factored into estimates of uncertainty; however, it should be considered by those interpreting estimates of live carbon stores and fluxes generated from national or local inventory based accounting protocols, especially in applications, such as valuing carbon credits, where unbiased estimates are critical. Model-selection uncertainty is not an easily-remedied error and may call into question the premise of being able to track forest carbon with the precision and accuracy required to support contemplated offset protocols. Our analysis indicates that the only way to truly reduce uncertainty from model selection is to subdivide the existing biomass equations or to develop an equation form that can predict the range present in existing equations. Our analysis suggests that for the latter solution to succeed, addition of tree height will not work unless some information on tree form is also included.

## Methods

### Study area

**Figure 5:**
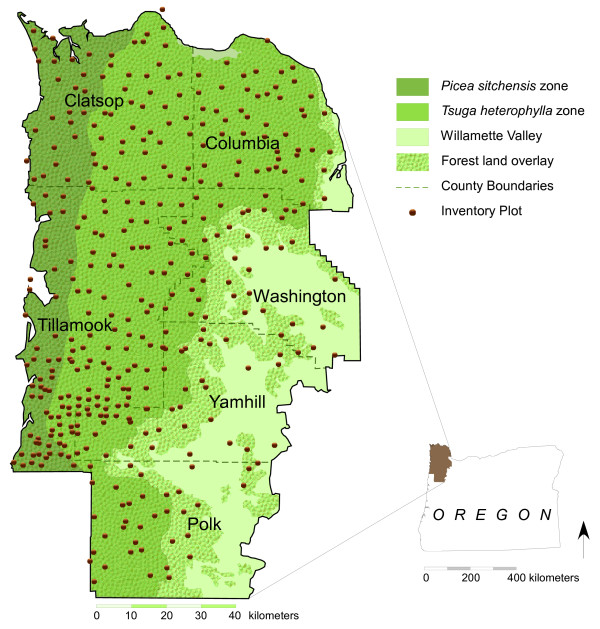
**Study area**. Northwest Oregon counties, forest land, vegetation zones, and Forest Service inventory plots. The *Picea sitchensis *zone boundary was derived from Franklin and Dyrness [33]; the Willamette Valley boundary was obtained from Level III Ecoregions of Oregon [43] and forest land coverage came from the Oregon Gap Analysis Program [44]. Inventory plots [12] are shown somewhat offset from their true locations to protect landowner privacy and to comply with applicable laws and regulations. Plot concentration on the Siskiyou National Forest (southern Tillamook and western Yamhill Counties) is greater because of greater sampling intensity on National Forest Land.

This study encompassed northwest Oregon (NWOR), defined here as the six counties in the northwest corner of Oregon: Clatsop, Columbia, Polk, Tillamook, Washington, and Yamhill. These counties cover 1.37 million ha, 65% of which is estimated by the Forest Inventory and Analysis Program (FIA; a nation wide program of the USDA Forest Service that conducts forest resource inventories throughout the US) to contain forest land [12] (which is defined as land having a current live-tree stocking or canopy cover of at least 10% or having had this in the past and having a high likelihood of having it in the future [7]).The counties fall in the Coast Range and Willamette Valley provinces [33] (Figure 5). The Coast Range is characterized by steep ridges and conifer-dominated forests and contains two major forest types: *Picea sitchensis *(Sitka spruce) and *Tsuga heterophylla *(western hemlock). Sitka spruce is found mainly in a coastal strip with high rainfall and mild temperatures; western hemlock is found in similar areas but with more variation in both precipitation and temperature. The Willamette Valley is inland and receives less precipitation and higher temperatures. Approximately 5% of the forest land area is administered by the U.S. Forest Service, 33% is administered by other public agencies, and the remaining 62% is privately owned [12].

### Tree species

We considered only the five most commonly occurring tree species in NWOR (the "target species" set) to avoid confounding effects from the high-degree of equation substitution employed for less common (and less frequently studied) species. The target species include three conifers: *Picea sitchensis *(Sitka spruce), *Pseudotsuga menziesii *(Douglas fir), and *Tsuga heterophylla *(western hemlock) and two hardwoods: *Acer macrophyllum *(bigleaf maple) and *Alnus rubra *(red alder), They collectively account for 90% of all live trees estimated by the forest inventory to exist in NWOR [12]. What we refer to hereafter as estimates of total live-tree C are, in fact, estimates of C in live trees of these 5 species.

### Sources and criteria for equation selection

We obtained relevant equations for volume and dry biomass from BIOPAK [18], the Jenkins et al. Comprehensive Database [34], and other available literature (see Additional files 1 and 2). Equations were deemed relevant if data originated, at least in part, from western British Columbia, Canada, southern coastal Alaska, or from the area west of the Cascade crest in OR and Washington (WA). However, some root and stump equations from the eastern U.S., Canada, and parts of Europe were included owing to the limited number of appropriate local equations for these components. Equations were excluded if they (1) relied on variables other than DBH and height (although equations relying on components we could calculate from DBH and height were allowed), (2) were not accompanied by the range of DBHs used to develop the equation, (the developmental DBH range; excepting the Weyerhaeuser stem wood volume equation [35] because it is used by FIA), (3) used stump heights other than 10, 15, or 30 centimeters (cm; the most common values, corresponding roughly to 4, 6, and 12 inches), (4) did not extend to the top of the stem (excepting equations from [26]).

### Height estimation

Some biomass- and almost all volume-prediction equations require height as an input variable. To obtain generic prediction envelopes based solely on DBH, we therefore generated height estimates for each species using the equation form(1)

where

h = total tree height in meters,

d = DBH outside bark in cm,

e = the base of natural logarithms, 2.71828...,

b_0 _= maximum height,

b_1 _= steepness parameter, and

b_2 _= curvature parameter [36].

This equation form is useful for its asymptotic behavior, which eliminates the unrealistic height estimates given by many other equation forms for large trees. It was created from mean measured height of trees in NWOR for each DBH class [12] weighted by DBH^-1 ^using nonlinear regression in SAS [37]. Parameters and standard errors for these regressions appear in Table 2.

**Table 2:** Northwest Oregon height equations

Species	**b**_**0 **_**(SE)**	**b**_**1 **_**(SE)**	**b**_**2 **_**(SE)**	n	MSE
*Picea sitchensis*	62.5163 (1.5675)	-0.0122 (0.00246)	1.0123 (0.0724)	121	25.11
	
*Pseudotsuga menziesii*	63.054 (2.9339)	-0.016 (0.00109)	1.0711 (0. 0338)	142	6.95
	
*Tsuga heterophylla*	53.7148 (2.2051)	-0.0175 (0.00242)	0.9956 (0.0536)	103	10.08
	
*Acer macrophyllum*	30.8836 (1.5888)	-0.0388 (0.00729)	0.9151 (0.0901)	80	15.70
	
*Alnus rubra*	32.2499 (2.1472)	-0.0271 (0.00558)	0.7346 (0.0489)	71	7.51

Coefficients for equation 1 in text. Equation form is .

### Calculation roadmap

**Figure 6:**
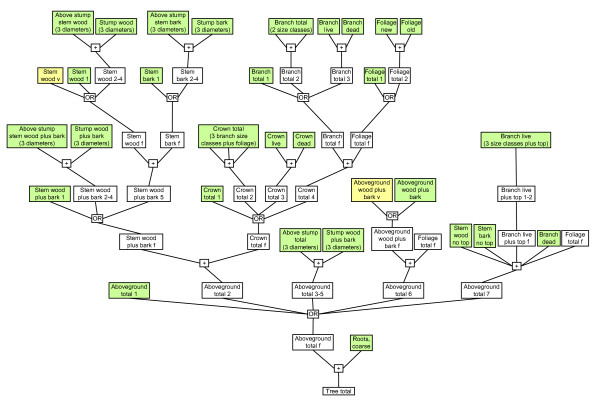
**Roadmap for component aggregation**. Generic calculation pathway for components of the target species. Component lookup table names appear in large rectangles. Green backgrounds indicate estimates were derived from biomass equations, then converted to C. White backgrounds denote lookup tables resulting from the aggregation process. Components with yellow backgrounds and a "v" following the component name are estimates from volume equations that were first multiplied by density to produce biomass estimates, then converted to C. Numbers after component names act as markers to separate lookup tables from different pathways. A range of numbers marks where a pathway was condensed to simplify the roadmap. Component names followed by "f" mark the final lookup table for a component after aggregation. Processing steps shown here in small boxes are addition (+) and comparison (OR). All steps were not performed for each species because prediction equations did not exist in every case.

There are few equations that predict total tree volume or biomass; instead equations predict lesser components and these values are combined to calculate volume or biomass for an entire tree. We defined a component as any single or aggregate part of a tree (e.g., small live branches are a component of more aggregated components, such as live crown and aboveground total). Given that researchers have developed equations for numerous, but not always standardized, components, we established a "road map" for estimating biomass via various calculation pathways (Figure 6). This map does not represent every calculation possibility because equations relying on variables other than DBH and height or using stump height and top diameters other than those mentioned previously were excluded. Some species do not have potentially suitable equations for every component in Figure 6, and for some components, there is only one potentially suitable equation.

### Prediction envelopes

We used the collected volume and biomass equations to create a total tree C "prediction envelope" for each species. This envelope encompassed the range of possible C values between the uppermost and lowermost predictions given by all possible combinations of the equations. To convert volume to biomass, we used density values from the literature (see Additional files 2 and 3) and retained the lowest and highest values for each species and component combination to create two biomass estimates based on each volume equation. Prediction envelopes were stored as lookup tables containing biomass ranges for each species and component by each 1-cm DBH class.

### Calculation approaches

Few equations have been developed using data that encompass the full range of DBHs present in NWOR. Extrapolation of equations beyond the developmental DBH range, although statistically invalid, has been unavoidable for anyone needing to obtain estimates for large trees; the FIA Program, for example, has many large trees in their sample, and in some areas, these account for much of the live tree C. We therefore examined three contrasting approaches: approach 1 used each equation over the entire species DBH range with no corrections; approach 2 used each equation only over its developmental DBH range; approach 3 used a combination of extrapolated and modified equations when required to produce "reasonable" estimates at all DBH classes.

Crown components, especially foliage, were not expected to increase significantly after a tree reached maturity, as assumed by Turner and Long [20]. Therefore we truncated crown component predictions for approach 3 in the middle of the species NWOR DBH range (as given in [12]) and applied predicted values at those points to all larger DBHs. However, the only species so modified for the purposes of this analysis was *Alnus rubra*, starting at the 54 cm DBH class; all other modifications began above 66 cm. Further details of approach 3 methods may be found in Additional file 4.

### Conversion of biomass to C

The C content of wood for the target species set ranged from 47.7 to 50.6% of dry biomass [38], although C content of other components might be significantly different for some species [39]. However, following Gifford's [39] suggestion of using 50 +/-2% for Australian national C estimates, biomass lookup table minima for all components were multiplied by 48% and the maxima by 52% for all species to account for the uncertainty in this conversion factor.

### Incorporation of possible correlation between tree components

Species-specific total tree C prediction envelopes were generated via addition and comparison of envelopes for all lesser tree components, following the sequences depicted in Figure 6. Because we summed ranges rather than point estimates, "correlation" between components could differentially affect the width of the envelopes at each addition step. We refer here not to statistically-calculated correlations, but to patterns that might occur as trees partition resources. Consider a hypothetical tree of a given diameter, which may have grown taller than others in its DBH class and so has a higher stem wood biomass. Being a larger tree, it might have more branch biomass and root biomass (positive correlation). On the other hand, trees are also known to allocate resources to one component at the expense of the others, so a taller stem might indicate less biomass in the branches and roots (a negative correlation). Correlation between all pairs of tree components is unknown, and likely varies, so we devised a method to examine sensitivity using two extreme examples of correlation. In the first, we assumed completely positive correlation at each addition step for all approaches. In the second, we assumed negative correlation at each addition step. We do not consider either option to be particularly realistic; however, we sought to bracket the possibilities, not find a most likely value. (Further methods and an example may be found in Additional file 4.)

### Applying prediction envelopes to inventory data

Total live-tree C prediction envelope values were linked with FIA inventory data [12] to produce a potential live-tree total tree C range for NWOR. Inventory data include tree measurements as well as necessary expansion factors for scaling plot data to county- and state wide levels. Appropriate prediction envelope bounds were then multiplied by expansion factors for each tree in the database. We summed the resulting values by species, then summed species totals to produce total live tree C storage bounds for NWOR. Total live tree C storage was calculated for both positive and negative correlation assumptions to assess how sensitive estimates were to correlation of tree components. Our reported uncertainty values represent half the output range and were also expressed as a percentage of the midpoint C estimate. Basing our uncertainty output on the midpoint or using the midpoint as a point of comparison between approaches is not meant to imply that it is the most likely value as this study was designed to examine the possible range of estimates caused by model selection.

### Comparison with other Pacific Northwest regional estimates

We compared our NWOR live-tree C ranges from approach 2 with single-point NWOR C estimates produced using biomass equations presented in several separate articles (which we label "single-source" estimates, even if the author(s) incorporated equations developed outside of their own study (e.g., [14])). Our prediction envelope approach, in contrast, produced what might be called "multiple-source" estimates. For each component at a given DBH, one equation became the lower, and one the upper, bound of our prediction envelope. However, owing to differing equation forms and coefficients, the same equations often were not the bounds over the entire DBH range of a prediction envelope; thus equations from multiple sources could contribute to the bounds of our final total tree C prediction envelope. Single-source estimates for tree total biomass were rare in the literature, but were abundant for aboveground total and stem wood plus bark. Such single-source estimates were not necessarily local equations; some were regional or even national, and a few were national multispecies equations (e.g., [13]). We undertook two comparisons: one limited to total tree, aboveground total, and stem wood plus bark of *Pseudotsuga menziesii *26 to 60 cm DBH to enable as many single-source estimates as possible, and the second limited to comparison between the FIA and Jenkins et al. [13] estimates but including all target species 3 to 66 cm DBH for tree total C.

Published and Web-posted FIA volume and biomass estimates are often relied upon as a basis for estimating biomass and C (e.g., [3,4,40]). However, PNW FIA biomass estimates lack foliage, dead branches, and any trees under 2.5 cm DBH. To compare FIA single-source estimates with our total tree and aboveground C, we added C from our prediction envelopes for missing tree components as a range at each DBH. No correction was made for small trees because our final DBH comparison range was constrained by the limitations imposed by approach 2 and did not extend to such low DBHs. All single-source biomass estimates used a 50% C-to-biomass conversion factor for this comparison only, excepting the components added to the FIA estimate.

To compare our NWOR totals with FIA-based estimates for the 3 to 66 cm DBH range for all target species, it was necessary to fill in some gaps in our prediction envelopes for branch dead, foliage total, and roots coarse with output from approach 3. The Jenkins et al. [13] tree totals also required limited extrapolation of their root equations over a few DBH classes for three species.

### Comparison with other estimates of error

Other estimates of error generally include only sampling error (as in FIA reports, e.g., [7]) and, more rarely, errors generated by measurement and regression [5]. To estimate FIA sampling error for NWOR live-tree C we examined the most recent FIA report for the western OR periodic inventory and obtained one standard error (SE) for a range of volume estimates [7]. Volume was multiplied by the average density (weighted based on stem wood volume in NWOR, using FIA densities [12] of the target species set to convert to biomass and a C:biomass conversion factor of 50% to obtain sampling error in C. An approximate 95% confidence interval for the appropriate C value was obtained by doubling the associated SE.

Diameter measurement variation is generally <2% of diameter (expressed as a 95% confidence interval; [41]). To obtain an approximation of the magnitude of diameter measurement error for FIA-reported volume, we followed the example of Phillips et al. [5] and took a simple equation form, calculated standard error from a 2% DBH measurement error for each tree in the database, applied expansion factors, and summed to the NWOR level. This assumed that a simple equation form was used for each tree, that there was no measurement error in height, and that errors were independent.

Jenkins et al. [13] reported bounds that contained 80% of their residuals for each multi-species equation. These residuals were from pseudodata, so they do not represent exactly what traditional regression residuals do, but we wished to see how such bounds would compare to the output of our analysis. As a quick test, we used the Jenkins et al. [13] NWOR live-tree C from our second comparison in the previous section and calculated a simple range for each species using their data, then summed the bounds for each species to the NWOR level. Their reported values only apply to their aboveground equations, but we applied them to the sum of the aboveground and root biomass equations.

### Strategies to reduce model-selection uncertainty

#### Comparison of Equation Forms

One way to reduce uncertainty would be to determine which, if any, equations were more accurate predictors. It is sometimes assumed that by accounting for height variation, so-called standard equations (those that incorporate both DBH and height as dependent variables) are more widely applicable than local (DBH-only) ones. We tested this by plotting *Pseudotsuga menziesii *stem wood biomass predicted by several standard equations (equation numbers 157, 204, 1536, 1932, and 2692 from [16,26,18,31], and [42]; see equations in Additional file 1) against the product of DBH^2 ^and height. We expected that if incorporation of height into the regression reduced uncertainty, standard equation predictions would converge more when plotted against the product of DBH^2 ^and height (a common variable in standard equations) than when plotted against only DBH or height.

#### Assigning Equations to Subpopulations

If volume and biomass equations could be accurately assigned to individual trees, uncertainty of the live-tree C estimate should decrease. We tested our knowledge about regression equation assignment by creating a scenario in which hypothetical equations were represented by dividing the total tree C envelope into sub-envelopes. Each hypothetical equation accounted for an equal proportion of the total tree C envelope and was assigned to an equal number of trees. For each species and DBH class, the total tree C envelope was divided into 2, 3, 4, 5, or 10 smaller envelopes of identical width, and trees were partitioned into 2, 3, 4, 5, or 10 equal-sized groups. Then we applied the appropriate number of trees to the new sets of C bounds for that species to obtain NWOR totals for each hypothetical equation, then summed the resulting minima and maxima to achieve the new NWOR live-tree C range.

## Competing interests

The authors declare that they have no competing interests.

## Authors' contributions

SLM performed the analysis, wrote computer programs to analyze prediction envelopes and apply them to the FIA database, and was a contributing author. MEH oversaw the research and was a contributing author. JSF provided advice on the FIA data and was a contributing author. JBD wrote the core programs that automate the production of equation predictions and the addition of prediction envelopes. All authors have read and approved the final manuscript.

## Supplementary Material

Additional file 1 **Excel spreadsheet of equations used in this study**. Click here for file

Additional file 2 **Word document of references for equations and density**. Click here for file

Additional file 3 Word document of densities used in this study. Click here for file

Additional file 4 PowerPoint presentation containing detailed methods and brief worked examples. Click here for file
