# Supplementary material for: Estimates of live-tree carbon stores in the Pacific Northwest are sensitive to model selection
Source: Carbon Balance Manag. 2011 Apr 10;6:2. doi: 10.1186/1750-0680-6-2 (PMC3094363; doi:10.1186/1750-0680-6-2)
Supplement: Additional file 2 — Word document of references for equations and density. [file 1750-0680-6-2-S2.DOC]

**Additional file 2: 2_Equation_and_density_references.doc.**

References for wood densities and volume and biomass equations. Author names that appear in Additional files 1, 3, and 4 may not appear here if we obtained the equation or density from the secondary source listed in the additional file. For example, we obtained density data, originally from Standish in 1983, from Gonzalez 1990 (cited in Additional file 3 as, “Standish 1983 in Gonzalez 1990”); only Gonzalez 1990 is included in this list.

**Reference List**

Alemdag IS: *Biomass of the Merchantable and Unmerchantable Portions of the Stem.* Inf Rep PI-X-20. Ontartio: Canadian Forestry Service, Petawawa National Forestry Institute; 1982.

Barclay HJ, Pang PC, Pollard DFW: **Aboveground biomass distribution within trees and stands in thinned and fertilized Douglas-fir.** *Can J For Res* 1986, **16:**438-442.

Baskerville GL: **Use of logarithmic regression in the estimation of plant biomass.** *Can J For Res* 1972, **2:**49-53.

Binkley D: **Ecosystem production in Douglas-fir plantations: interaction of red alder and site fertility.** *For Ecol Manage* 1983, **5:**215-227.

Bormann BT: **Diameter-based biomass regression models ignore large sapwood-related variation in Sitka spruce.** *Can J For Res* 1990, **20:**1098-1104.

Brackett M: *Notes on Tarif Tree Volume Computation.* Resour Manage Rep No. 24. Olympia: State of Washington Department of Natural Resources; 1977.

Browne JE: *Standard Cubic-Foot Volume Tables for the Commercial Tree Species of British Columbia, 1962.* Vancouver: British Columbia Forest Service Forest Surveys and Inventory Division; 1962.

Bruce D, DeMars DJ: *Volume Equations for Second-Growth Douglas-Fir*. Res Note PNW-239. Portland: USDA Forest Service Pacific Northwest Forest and Range Experiment Station; 1974.

Chambers CJJr, Foltz BW: *The Tarif System -- Revisions and Additions.* DNR Note No. 27. Olympia: Washington State Department of Natural Resources; 1979.

Curtis RO, Bruce D, VanCoevering C: *Volume and Taper Tables for Red Alder*. Res Pap PNW-56. Portland: USDA Forest Service Pacific Northwest Forest and Range Experiment Station; 1968.

Espinosa Bancalari MA, Perry DA: **Distribution and increment of biomass in adjacent young Douglas-fir stands with different early growth rates.** *Can J For Res* 1987, **17:**722-730.

Feller MC: **Generalized versus site-specific biomass regression equations for *Pseudotsuga menziesii* var. *menziesii*  and *Thuja plicata* in coastal British Columbia.** *Bioresour Technol* 1992, **39:**9-16.

Franklin J: **Dendrometer studies for stand volume and height measurements: Long-Term Ecological Research.** [database]. Forest Science Data Bank: TV009 [http://www.fsl.orst.edu/lter/data/abstract.cfm?dbcode=TV009]

Gholz HL, Grier CC, Campbell AG, Brown AT: *Equations for Estimating Biomass and Leaf Area of Plants in the Pacific Northwest.* Res Pap 41. Corvallis: Oregon State University Forest Research Laboratory; 1979.

Gonzalez JS: *Wood Density of Canadian Tree Species.* Inf Rep NOR-X-315. Edmonton: Forestry Canada, Northwest Region, Northern Forestry Centre; 1990.

Grier CC, Lee KM, Archibald RM: **Effect of urea fertilization on allometric relations in young Douglas-fir trees.** *Can J For Res* 1984, **14:**900-904.

Grier CC, Logan RS: **Old-growth *Pseudotsuga menziesii* communities of a western Oregon watershed: biomass distribution and production budgets.** *Ecol Monogr* 1977, **47:** 373-400.

Hann DW: *A Key to the Literature Presenting Tree Volume and Taper Equations for Species in the Pacific Northwest and California.* Res Contrib 6. Corvallis: Oregon State University Forest Research Laboratory; 1994.

Harmon ME, Garman SL, Ferrell WK: **Modeling historical patterns of tree utilization in the Pacific Northwest: carbon sequestration implications.** *Ecol Appl* 1996, **6:**641-652.

Helgerson OT, Cromack K, Stafford S, Miller RE, Slagle R: **Equations for estimating aboveground components of young Douglas-fir and red alder in a coastal Oregon plantation.** *Can J For Res* 1988, **18:**1082-1085.

Hiserote B, Waddell K: *PNWFIA IDB: the integrated database* *1.4* [MS Access database]. Portland: USDA Forest Service Pacific Northwest Research Station; 2004.

Hoyer GE: *Tree Form Quotients As Variables in Volume Estimation*. Res Pap PNW-345. Portland: USDA Forest Service Pacific Northwest Forest and Range Experiment Station; 1985.

Jenkins JC, Chojnacky DC, Heath LS, Birdsey RA: **National-scale biomass estimators for United States tree species.** *For Sci* 2003, **49:**12-35.

Jenkins JC, Chojnacky DC, Heath LS, Birdsey RA: *Comprehensive Database of Diameter-Based Biomass Regressions for North American Tree Species*. Gen Tech Rep GTR-NE-319. Newtown Square: USDA Forest Service Northeastern Research Station; 2004.

Kurucz J: **Component weights of Douglas-fir, western hemlock, and western red cedar biomass for simulation of amount and distribution of forest fuels.** *MS thesis.* University of British Columbia, Forestry Department; 1969.

Le Goff N, Ottorini J-M: **Root biomass and biomass increment in a beech (*Fagus sylvatica* L.) stand in north-east France.** *Ann For Sci* 2001, **58:**1-13.

Long JN, Turner J: **Aboveground biomass of understory and overstory in an age sequence of four Douglas-fir stands.** *J Appl Ecol* 1975, **12:**179-188.

Marshall PL, Wang Y: *Above Ground Tree Biomass of Interior Uneven-Aged Douglas-Fir Stands.* University of British Columbia Working Paper WP-1.5-003. Canada-British Columbia Partnership Agreement on Forest Resource Development; 1995.

Means JE, Hansen HA, Koerper GJ, Alaback PB, Klopsch MW: *Software for Computing Plant Biomass–BIOPAK Users Guide*. Gen Tech Rep PNW-GTR-340. Portland: USDA Forest Service Pacific Northwest Research Station; 1994.

Omule SAY, Fletcher VE, Polsson KR: *Total and Merchantable Volume Equations for Small Coastal Douglas-Fir.* FRDA Rep 010. Victoria: Canadian Forestry Service and BC Ministry of Forests and Lands; 1987.

Pillsbury NH, Kirkley ML: *Equations for Total, Wood, and Saw-Log Volume for Thirteen California Hardwoods*. Res Note PNW-414. Portland: USDA Forest Service Pacific Northwest Forest and Range Experiment Station; 1984.

Raile GK: *Estimating Stump Volume*. Res Pap NC-224. St. Paul: USDA Forest Service North Central Forest Experiment Station; 1982.

Sachs D: **Management effects on nitrogen nutrition and long-term productivity of western hemlock stands: an exercise in simulation with FORCYTE.** *MS thesis.* Oregon State University; 1983.

Santantonio D, Hermann RK, Overton WS: **Root biomass studies in forest ecosystems.** *Pedobiologia* 1977, **17:**1-31.

Shaw DL: **Biomass equations for Douglas-fir, western hemlock, and red cedar in Washington and Oregon.** In *Forest Resource Inventories*. Edited by Frayer WE. Fort Collins: Colorado State University; 1979:763-781.

Simpson W, Ten Wolde A: **Physical properties and moisture relations of wood.** In *Wood Handbook: Wood As an Engineering Material*. Gen Tech Rep FPL-GTR-113. Edited by Forest Products Laboratory. Madison: USDA Forest Service Forest Products Laboratory; 1999:11-14.

Smith JHG, Kozack A: **Thickness, moisture content, and specific gravity of inner and outer bark of some pacific northwest trees.** *For Prod J* 1971, **21:**38-40.

Snell JAK, Anholt BF: *Predicting Crown Weight of Coast Douglas-Fir and Western Hemlock*. Res Pap PNW-281. Portland: USDA Forest Service Pacific Northwest Forest and Range Experiment Station; 1981.

Snell JAK, Little SN: *Predicting Crown Weight and Bole Volume of Five Western Hardwoods*. Gen Tech Rep PNW-151. Portland: USDA Forest Service Pacific Northwest Forest and Range Experiment Station; 1983.

Snell JAK, Max T: *Estimating the Weight of Crown Segments for Old-Growth Douglas-Fir and Western Hemlock*. Res Pap PNW-329. Portland: USDA Forest Service Pacific Northwest Forest and Range Experiment Station; 1985.

St.Clair JB: **Family difference in equations for predicting biomass and leaf area in Douglas-fir (*Pseudotsuga menziesii var. menziesii*).** *For Sci* 1993, **39:**743-755.

Standish JT, Manning GH, Demaerschalk JP: *Development of Biomass Equations for British Columbia Tree Species.*  Rep BC-X-264. Vancouver: Canadian Forestry Service Pacific Forest Research Centre; 1985.

Stanek W, State D: *Equations Predicting Primary Productivity (Biomass) of Trees, Shrubs and Lesser Vegetation Based on Current Literature.* Rep BC-X-183. Victoria: Canadian Forestry Service Pacific Forest Research Centre; 1978.

Thies WG, Cunningham PG: **Estimating large-root biomass from stump and breast-height diameters for Douglas-fir in western Oregon.** *Can J For Res* 1996, **26:**237-243.

Ung C-H, Bernier P, Guo X-J: **Canadian national biomass equations: new parameter estimates that include British Columbia data.** *Can J For Res* 2008, **38:**1123-1132.

Walters DK, Hann DW, Clyde MA: *Equations and Tables Predicting Gross Total Stem Volumes in Cubic Feet for Six Major Conifers of Southwest Oregon.* Corvallis: Oregon State University, Forest Research Laboratory; 1985.

Zavitkovski J, Stevens RD: **Primary productivity of red alder ecosystems.** *Ecology* 1972, **53:**235-242.
