# Supplementary material for: Estimates of live-tree carbon stores in the Pacific Northwest are sensitive to model selection
Source: Carbon Balance Manag. 2011 Apr 10;6:2. doi: 10.1186/1750-0680-6-2 (PMC3094363; doi:10.1186/1750-0680-6-2)
Supplement: Additional file 3 — Word document of densities used in this study. [file 1750-0680-6-2-S3.DOC]

## Additional file 3: 3_Densities.doc

| **Species** | **Component** | **Low density** | **High density** | **Source/s for low density** | **Source for high density** |
| --- | --- | --- | --- | --- | --- |
|  |  | kg m^-3^ | kg m^-3^ |  |  |
| *Picea sitchensis* | wood | 330 | 412 | Simpson and TenWolde 1999 | Standish 1983 in Gonzales 1990 |
| *Pseudotsuga menziesii* | wood | 421 | 460 | Standish 1983 in Gonzales 1990 | Littleford 1961 in Gonzales 1990 |
| *Tsuga heterophylla* | wood | 380 | 436 | Krahmer 1966 in Gonzales 1990,  Markwardt and Wilson 1935 in Gonzales 1990 | Standish 1983 in Gonzales 1990 |
| *Acer macrophyllum* | wood | 440 | 466 | Simpson and TenWolde 1999,  Markwardt and Wilson 1935 in Gonzales 1990 | Jessome 1977 in Gonzales 1990 |
| *Alnus rubra* | wood | 370 | 395 | Simpson and TenWolde 1999,  Markwardt and Wilson 1935 in Gonzales 1990 | Standish 1983 in Gonzales 1990 |
| Note: High- and low-density values from the literature for northwest Oregon target species, used in conversion of wood volume to biomass. These values are usually averages of multiple samples; they do not reflect the full range of densities occurring in individual wood samples. References are in Additional file 2. | | | | | |
